# Association of cerebrospinal fluid amphiphysin-1 levels with cognition and Alzheimer’s disease pathology biomarkers

**DOI:** 10.3389/fnagi.2026.1875646

**Published:** 2026-07-21

**Authors:** Jia-De Fan, Yan Fu, Qiong-Yao Li, Zi-Qi Zhang, Yong-Li Zhao, Quan Hao, Lan Tan, Meng-Shan Tan, the Alzheimer’s Disease Neuroimaging Initiative

**Affiliations:** 1 Department of Neurology, Qingdao Municipal Hospital, Qingdao University, Qingdao, China; 2 School of Clinical Medicine, Shandong Second Medical University, Weifang, China; 3 Department of Neurology, Qingdao Municipal Hospital, University of Health and Rehabilitation Sciences, Qingdao, China; 4 Department of Neurology, Qingdao Municipal Hospital, Dalian Medical University, Qingdao, China

**Keywords:** Alzheimer’s disease, Amphiphysin-1, biological markers, cerebrospinal fluid, cognition, synapses

## Abstract

**Introduction:**

Amphiphysin-1 (AMPH), an accessory component of the clathrin-mediated endocytosis (CME) machinery, plays a critical role in synaptic vesicle recycling and membrane dynamics and has been implicated in Alzheimer’s disease (AD) risk. However, its relationship with core AD biomarkers and disease progression remains unclear.

**Methods:**

In this study, we examined the associations between cerebrospinal fluid (CSF) AMPH and core AD biomarkers (CSF amyloid-β42 [Aβ_42_], phosphorylated tau [P-tau], and total tau [T-tau]), cognitive performance, neurodegeneration, and clinical progression in 723 participants from the Alzheimer’s Disease Neuroimaging Initiative (ADNI). Participants were categorized according to the AT(N) framework into stage 0, stage 1, stage 2, and suspected non-AD pathophysiology (SNAP).

**Results:**

AMPH exhibited stage-dependent alterations across the AD continuum, characterized by lower levels in stage 1, and elevated levels in stage 2 and SNAP. Cross-sectionally, CSF AMPH was positively associated with core CSF biomarkers. Longitudinal analyses showed that, among amyloid-negative individuals, higher baseline AMPH was associated with faster decreases in CSF Aβ_42_ and increases in P-tau and T-tau. In the overall cohort, higher baseline AMPH was associated with accelerated hippocampal atrophy and cognitive decline, and with an increased risk of clinical progression (HR = 1.21, 95% CI: 1.05–1.37).

**Discussion:**

In ADNI, CSF AMPH was associated with biological changes across the AD continuum, cognitive decline, hippocampal atrophy, and clinical progression. These findings may provide complementary information for understanding disease progression.

## Introduction

Alzheimer’s disease (AD) is a chronically progressive neurodegenerative condition characterized by worsening cognition, synaptic impairment, and extensive neuronal degeneration (Knopman et al., 2021). Although extracellular amyloid-β (Aβ) deposits and intraneuronal aggregates of hyperphosphorylated tau are well-established pathological lesions and central hallmarks of AD, pathological changes can develop silently for 15–20 years before symptoms become clinically evident (Ising et al., 2015; Thal et al., 2014). This long preclinical phase underscores the need to identify additional biomarkers and mechanisms beyond amyloid and tau pathology. Emerging evidence has implicated clathrin-mediated endocytosis (CME) in AD pathogenesis (Jaye et al., 2024; Wu and Yao, 2009). Genome-wide association studies have implicated CME-related genes, such as BIN1 and PICALM, as key contributors to AD susceptibility (Ando et al., 2020, 2022; Szabo et al., 2022). Experimental studies have further shown that CME regulates amyloid precursor protein (APP) internalization and Aβ generation and may also influence tau propagation (Koo and Squazzo, 1994; Wu and Yao, 2009).

Although CME research has largely focused on its core components, emerging evidence suggests that alterations in accessory proteins could substantially influence CME function, particularly in neurons (McMahon and Boucrot, 2011). Among these accessory proteins, Amphiphysin-1 (AMPH) appears particularly relevant to AD. AMPH, a critical CME accessory protein, promotes vesicle scission by sensing membrane curvature via its BAR domain and recruiting dynamin via its SH3 domain (Peter et al., 2004; Takei et al., 1999). Recent clinical studies have shown that AMPH expression is markedly decreased in the temporal and frontal cortices (De Jesús-Cortés et al., 2012). In the JNPL3 mouse model, which is characterized by pathological tau overexpression (Lewis et al., 2001), brain regions with substantial tau aggregation show markedly reduced AMPH protein levels (De Jesús-Cortés et al., 2012). AMPH-knockout mice exhibit severe neuropathological features, including synaptic dysfunction, seizures, and learning impairments (Di Paolo et al., 2002). Although AMPH expression appears reduced in brain tissue across human and animal studies, CSF AMPH levels have conversely been reported to be elevated in AD patients (Bergström et al., 2021). Together, these findings suggest that CSF AMPH may reflect AD-related processes, but its relationship with established biomarkers and disease progression remains unclear. On the basis of prior studies (Bergström et al., 2021; Remnestål et al., 2021), AMPH was prespecified before the current analyses as a hypothesis-driven candidate protein in the present study.

Therefore, we aimed to (1) compare CSF AMPH levels across groups defined by clinical diagnosis and AT(N) biomarker profiles; (2) evaluate associations between CSF AMPH and core AD biomarkers (CSF amyloid-β_42_ [Aβ_42_], phosphorylated tau [P-tau], and total tau [T-tau]); and (3) examine associations between CSF AMPH and cognitive performance, as well as whether baseline CSF AMPH was associated with longitudinal cognitive change and the risk of clinical progression.

## Materials and methods

### Study participants

Data were obtained from the Alzheimer’s Disease Neuroimaging Initiative (ADNI). ADNI is a public–private partnership launched in 2003 that longitudinally follows cognitively normal, MCI, and AD dementia participants, with the systematic collection of MRI/PET imaging, biomarker, and clinical/neuropsychological data.

A total of 723 ADNI participants were included in this study. Eligibility required available baseline CSF AMPH measurements, core AD biomarkers, complete demographic information, Apolipoprotein E (APOE) ε4 carriage status, and a clinical diagnosis. The sample included 171 cognitively normal (CN), 400 mild cognitive impairment (MCI), and 152 AD participants based on ADNI clinical diagnostic criteria.

### Measurements of AMPH, CSF biomarkers, and imaging

Cerebrospinal fluid AMPH data were obtained using the SomaScan 7K v4.1 platform, with normalization and quality control performed according to established protocols as previously reported (Wang et al., 2025). CSF Aβ_42_, P-tau, and T-tau levels were quantified with the Elecsys platform, as previously reported for ADNI (Bittner et al., 2016). Magnetic resonance (MR) images were obtained on 1.5T or 3.0T scanners with site-specific optimized acquisition protocols (Jack et al., 2008). For each participant, left and right hippocampal volumes were averaged.

### Cognitive measures

Memory and executive function were measured using ADNI composite memory score (ADNI-MEM) and executive function score (ADNI-EF; Crane et al., 2012). Global cognition was evaluated using the Alzheimer’s Disease Assessment Scale (ADAS-11, ADAS-13; Podhorna et al., 2016), Mini-Mental State Examination (MMSE; Folstein et al., 1975), Montreal Cognitive Assessment (MoCA; Nasreddine et al., 2005), and Clinical Dementia Rating (CDR; Lanctôt et al., 2024). Higher CDR and ADAS-11/ADAS-13 scores and lower MMSE, MoCA, ADNI-MEM, and ADNI-EF scores were interpreted as poorer cognitive performance.

### Group classification

Building upon the previous study (Rauchmann et al., 2021), we used the following CSF cutoffs to define AT(N) status: Aβ_42_ < 980 pg/mL for amyloid positivity (A+), P-tau > 24 pg/mL for tau positivity (T+), and T-tau > 266 pg/mL for neurodegeneration positivity (N+). According to the National Institute on Aging-Alzheimer’s Association (NIA-AA) criteria (Jack et al., 2018), participants were then grouped into stage 0 (A−T−N−), stage 1 (A+T−N−), stage 2 (A+T+N−, A+T−N+, A+T+N+), and suspected non-AD pathophysiology (SNAP). Participants were additionally stratified by Apolipoprotein E (APOE) ε4 carrier status (non-carrier or carrier).

### Statistical analysis

We reported continuous variables as mean (SD) and categorical variables as N (%). We excluded values >3 SDs prior to analysis. We evaluated normality with the Shapiro–Wilk test. Non-normally distributed variables were Box–Cox transformed (Supplementary Table 1) and subsequently z-standardized. We tested group differences using one-way ANOVA (normal continuous variables), Kruskal–Wallis tests (non-normal continuous variables), and chi-square tests (categorical variables). Differences in z-standardized CSF AMPH were assessed using Student’s *t*-tests for two groups and ANOVA followed by Tukey’s *post-hoc* test for three or more groups.

Primary hypothesis driven analyses examined CSF AMPH levels across diagnostic groups and AT(N) defined stages of the AD continuum and assessed cross sectional associations of CSF AMPH with core AD biomarkers, cognitive performance, and neuroimaging measures, as well as associations of baseline CSF AMPH with longitudinal changes in these measures and the risk of clinical progression. Interaction and subgroup analyses were designated as exploratory. Cross-sectional group comparisons were performed in the full sample. Regression and longitudinal analyses were restricted to CN and MCI participants. Multiple linear regression was used to assess associations of AMPH levels with core AD biomarkers, neuroimaging measures, and cognitive performance. Linear mixed effects models were used to assess the associations between baseline AMPH levels and longitudinal changes in core AD biomarkers, neuroimaging measures, and cognition outcomes. Follow up time was defined as years since baseline. Each model included baseline AMPH, standardized follow up time, the AMPH × time interaction, and prespecified covariates as fixed effects. Participant specific random intercepts and random slopes for time were included to account for within participant correlations across repeated measurements. The random intercept and random slope were allowed to correlate under an unstructured covariance matrix. The AMPH × time interaction term was used to assess whether baseline AMPH was associated with the longitudinal rate of change in each outcome. Interaction analyses were evaluated within the MLR and LMM frameworks to test effect modification by age, sex, and *APOE ε4* status. Subgroup analyses were subsequently conducted according to the interaction results.

Restricted cubic spline functions of baseline CSF AMPH were fitted in adjusted Cox proportional hazards models, with four knots placed at the 5th, 35th, 65th, and 95th percentiles of the CSF AMPH distribution. Adjusted hazard ratios and 95% confidence intervals across the distribution of baseline CSF AMPH were estimated using the median baseline CSF AMPH level as the reference value. *P*-values for the overall association and nonlinearity were calculated. AMPH levels were categorized into tertiles; individuals in the highest tertile (T3) were defined *a priori* as the high group, and those in the lower two tertiles (T1–T2) were defined as the low group. Sensitivity analyses additionally evaluated CSF AMPH categorizations defined by medians and quartiles. Clinical progression was defined as conversion from CN to MCI, or progression from MCI to AD. Kaplan–Meier curves were applied to compare the cumulative incidence of clinical progression (progress from CN to MCI, or MCI to AD) during follow-up across groups stratified by AMPH levels. Furthermore, Cox proportional hazards models were applied to evaluate the association between CSF AMPH and clinical progression. The proportional hazards assumption was verified using Schoenfeld residuals and was satisfied. To assess the incremental prognostic value of CSF AMPH, nested Cox models for clinical progression were fitted. Model 1 included age, sex, education, APOE ε4 status, and baseline diagnosis; Model 2 additionally included CSF Aβ_42_ and P-tau; and Model 3 further included CSF AMPH. Model fit was compared using likelihood ratio tests and Nagelkerke pseudo R^2^ values. All models were adjusted for age, sex, education, and APOE ε4 status. For analyses involving core AD biomarkers and neuroimaging measures, baseline diagnosis was additionally included as a covariate (additionally adjusted for intracranial volume in hippocampal volume models).

To account for multiple testing, the Benjamini Hochberg (BH) procedure was applied to control the false discovery rate (FDR) within each prespecified analytic domain, including cross sectional biomarker analyses, longitudinal biomarker analyses, cognitive outcomes, interaction analyses, and survival analyses. Both nominal *P*-values and BH adjusted *P*-values are reported. Statistical significance was defined as a two sided BH adjusted *P*-value < 0.05. Statistical analyses were performed using R (v4.1.1), including the survival, ggplot2, and dplyr packages.

## Results

### Participant characteristics

After strict quality control, 723 participants were included in the analysis, comprising 171 CN individuals, 400 MCI individuals, and 152 AD individuals. The mean age was 73.5 (±7.5) years, and mean education was 15.91 (±2.85) years. A total of 310 participants (42.8%) were female. Significant differences across the three groups were observed in age, APOE ε4 status, MMSE and ADAS-13 scores, as well as CSF AMPH, CSF Aβ_42_, CSF P-tau, and CSF T-tau levels; however, no significant differences were found for sex or years of education (Table 1).

**TABLE 1:** Characteristics of participants in ADNI.

Characteristics	CN (*n* = 171)	MCI (*n* = 400)	AD (*n* = 152)	Total (*n* = 723)	*P*-value
Age, years (mean ± SD)	74.60 (5.98)	72.20 (7.55)	75.41 (8.32)	73.56 (7.50)	**<0.001**
Female gender (*N*, %)	80 (46.7)	169 (42.2)	61 (40.1)	310 (42.8)	0.449
Education, years (mean ± SD)	16.30 (2.70)	15.90 (2.88)	15.42 (2.86)	15.91 (2.85)	0.176
*APOE ε4* carriers (*N*, %)	43 (25.1)	207 (51.7)	106 (69.7)	356 (49.2)	**<0.001**
MMSE (mean ± SD)	29.03 (1.34)	27.70 (3.31)	22.55 (3.71)	26.95 (3.23)	**<0.001**
ADAS-13 (mean ± SD)	9.81 (4.58)	16.50 (6.78)	30.42 (8.38)	17.72 (9.65)	**<0.001**
AMPH (RFU) (mean ± SD)	596.01 (158.27)	629.00 (196.71)	663.20 (185.30)	628.39 (186.99)	**0.005**
Aβ_42_ (pg/mL) (mean ± SD)	1347.84 (617.88)	1012.04 (540.33)	661.16 (366.35)	1017.69 (576.11)	**<0.001**
P-tau (pg/mL) (mean ± SD)	21.81 (8.84)	28.1 (14.95)	36.04 (14.83)	28.27 (14.51)	**<0.001**
T-tau (pg/mL) (mean ± SD)	237.97 (83.47)	289.00 (132.80)	362.14 (133.94)	292.35 (129.88)	**<0.001**

Categorical variables were reported as number and percentage; continuous variables were reported as means (SD). *P*-values were computed with the one-way ANOVA or Kruskal–Wallis test for continuous variables, and the χ^2^ test for categorical variables. Significant effects (*P* < 0.05) are shown in bold. APOE, Apolipoprotein E; MMSE, Mini-Mental State Examination; ADAS-13, Alzheimer’s Disease Assessment Scale–Cognitive Subscale 13; AMPH, Amphiphysin1; Aβ_42_, amyloid-β42; P-tau, phosphorylated-tau; T-tau, total-tau.

### Levels of CSF AMPH in different diagnostic groups

**FIGURE 1:**
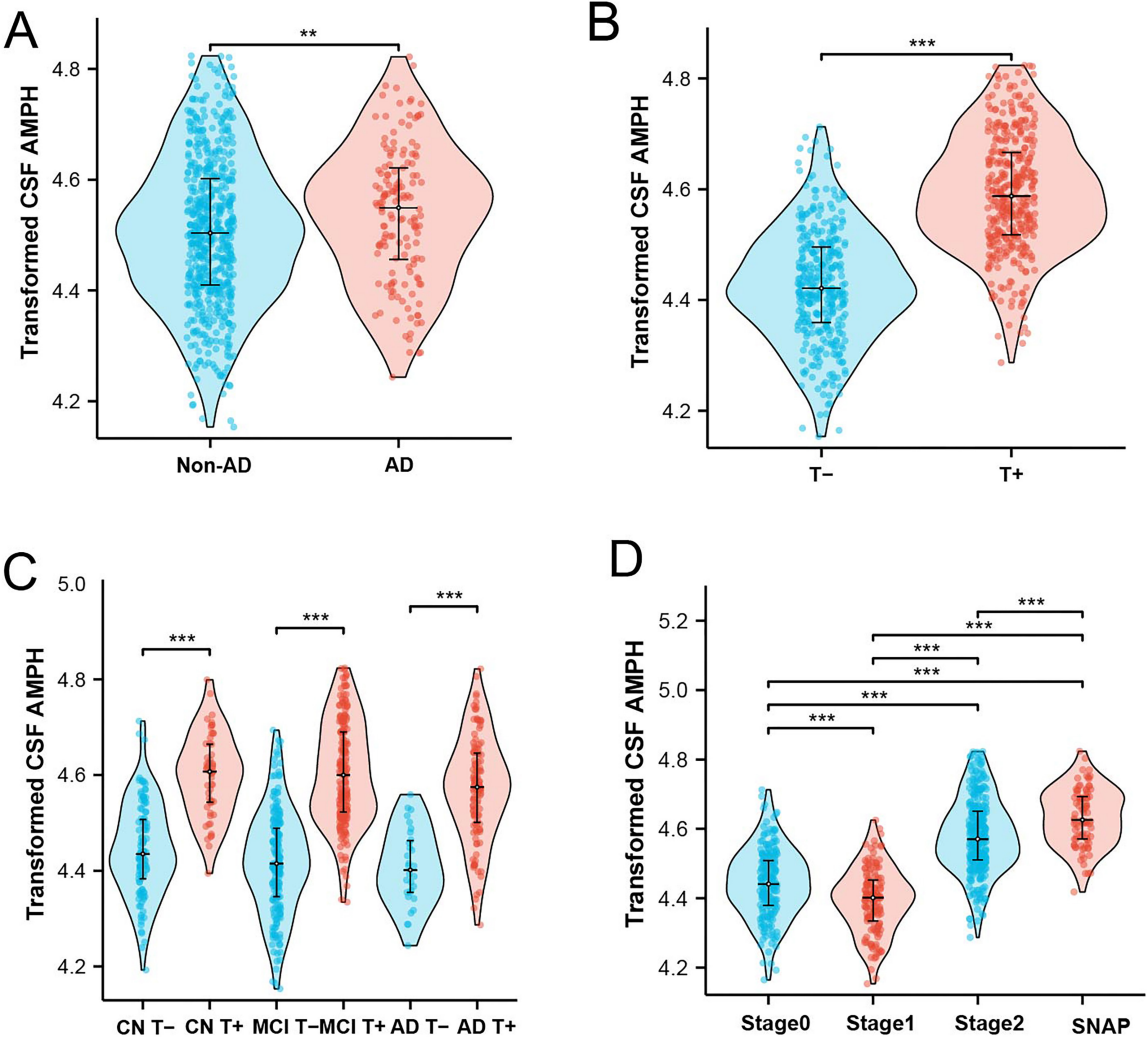
Transformed baseline CSF AMPH in participants classified according to clinical diagnosis and NIA-AA criteria. Transformed CSF AMPH levels were higher in individuals with AD than in non-AD participants **(A)**, higher in T+ than in T– participants **(B)**, consistently higher in T+ participants within each diagnostic group **(C)**, and were lowest in stage 1, whereas both stage 2 and SNAP exhibited higher levels **(D)**. Transformed CSF AMPH levels were compared across groups using one-way ANOVA, with Tukey’s HSD test applied for *post hoc* comparisons. A+ was defined as CSF Aβ_42_ < 980 pg/mL, T+ as CSF P-tau > 24 pg/mL, and N+ as CSF T-tau > 266 pg/mL. Significant effects ***P < 0.001; **P < 0.01; *P < 0.05. CSF, cerebrospinal fluid; AMPH, amphiphysin-1; CN, cognitively normal; MCI, mild cognitive impairment; AD, Alzheimer’s disease; T–, tau negative; T+, tau positive; SNAP, suspected non-Alzheimer’s pathophysiology; NIA-AA, National Institute on Aging–Alzheimer’s Association.

Baseline CSF AMPH levels differed significantly across clinical diagnostic groups. Compared with the non-AD groups, the AD group had higher CSF AMPH levels (Figure 1A). T+ individuals exhibited elevated CSF AMPH concentrations compared with T− individuals (*P* < 0.001; Figure 1B). Across the CN, MCI, and AD groups, T+ participants consistently showed higher CSF AMPH levels than T− participants (all *P* < 0.001; Figure 1C). CSF AMPH levels were lower in stage 1 and elevated in stage 2 and SNAP (all *P* < 0.001; Figure 1D). In addition, CSF AMPH levels were significantly higher in N+ individuals (*P* < 0.001). No significant differences were observed between the A+ and A− groups (Supplementary Table 2).

### Associations between CSF AMPH and core CSF biomarkers

**FIGURE 2:**
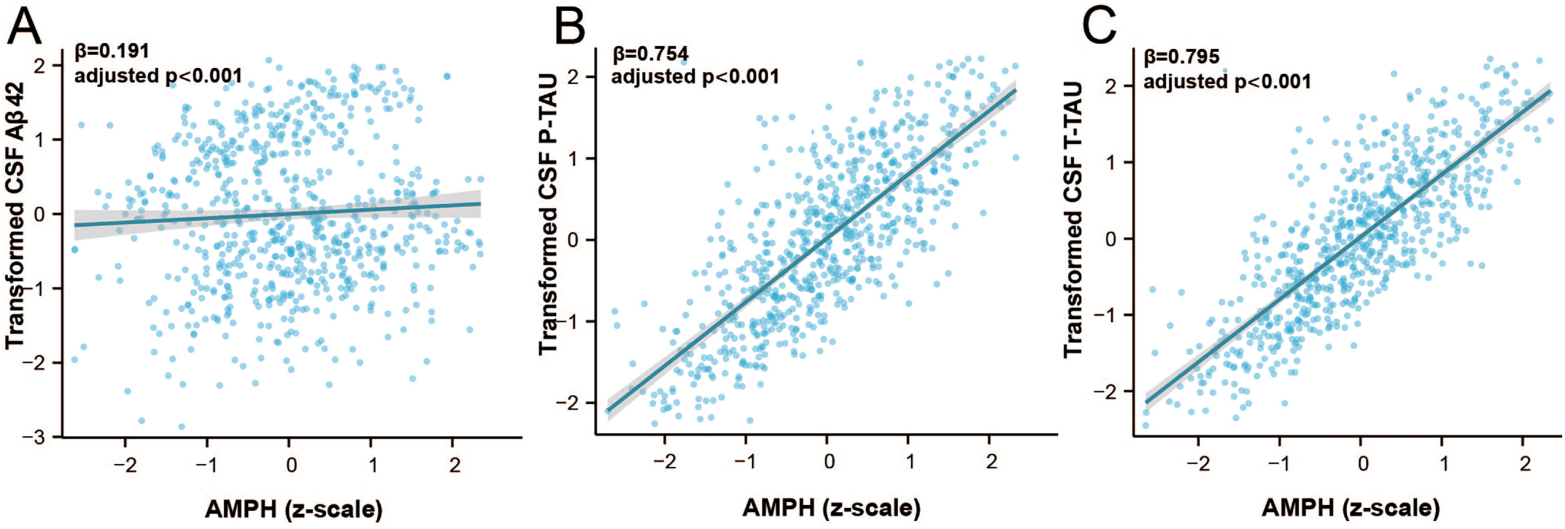
Associations between baseline CSF AMPH and core CSF biomarkers. CSF AMPH levels were positively associated with CSF Aβ_42_ **(A)**, P-tau **(B)**, and T-tau **(C)**. The normalized regression coefficients (β) and *P*-values were derived from multiple linear regression adjusted for age, sex, education, baseline diagnosis, and *APOE ε4* status. Abbreviations: CSF, cerebrospinal fluid; AMPH, amphiphysin-1; P-tau, phosphorylated tau; T-tau, total tau; Aβ_42_, amyloid-β42; APOE, Apolipoprotein E.

Across the full cohort, CSF AMPH levels showed positive associations with CSF Aβ_42_ (β = 0.191, *P* < 0.001, adjusted *P* < 0.001), P-tau (β = 0.754, *P* < 0.001, adjusted *P* < 0.001), and T-tau (β = 0.795, *P* < 0.001, adjusted *P* < 0.001) (Figures 2A–C). By contrast, no association was observed between CSF AMPH and hippocampal volume (β = 0.066, *P* = 0.087, adjusted *P* = 0.087).

### Association of baseline CSF AMPH with longitudinal changes in CSF core biomarkers and neuroimaging measures

In the overall sample, baseline CSF AMPH was associated with hippocampal atrophy over follow-up (β = −0.023, *P* < 0.001, adjusted *P* < 0.001), whereas no significant associations were observed for longitudinal changes in CSF Aβ_42,_ P-tau or T-tau (Table 2). When stratified by amyloid status, higher baseline CSF AMPH levels among A− participants were associated with faster longitudinal decreases in CSF Aβ_42_ (β = −0.049, *P* < 0.001, adjusted *P* < 0.001), as well as more rapid increases in CSF P-tau (β = 0.026, *P* < 0.001, adjusted *P* < 0.001) and T-tau (β = 0.024, *P* = 0.001, adjusted *P* < 0.001) (Table 2). In contrast, baseline CSF AMPH was not significantly related to longitudinal changes in CSF Aβ_42_, P-tau, or T-tau among A+ individuals (Table 2). Notably, baseline CSF AMPH remained significantly associated with hippocampal atrophy in both A− (β = −0.020, *P* < 0.001, adjusted *P* < 0.001) and A+ (β = −0.025, *P* < 0.001, adjusted *P* < 0.001) groups (Table 2).

**TABLE 2:** Associations of baseline CSF AMPH levels with longitudinal changes in CSF biomarkers and hippocampal volume by amyloid status.

	A−			A+			Overall		
Longitudinal measures	β	*P*	Adjusted P	β	*P*	Adjusted P	β	*P*	Adjusted *P*
CSF Aβ_42_	−0.049	<0.001	**<0.001**	0.017	0.133	0.264	−0.013	0.039	0.076
CSF P-tau	0.026	<0.001	**<0.001**	−0.008	0.341	0.359	0.009	0.078	0.105
CSF T-tau	0.024	0.001	**<0.001**	−0.006	0.359	0.359	0.007	0.166	0.166
Hippocampal volume	−0.020	<0.001	**<0.001**	−0.025	<0.001	**<0.001**	−0.023	<0.001	**<0.001**

The normalized regression coefficients (β) and *P*-values were derived from the interaction term of CSF AMPH × time in the linear mixed-effects models adjusted for age, sex, education, baseline diagnosis, *APOE ε4* status and intracranial volume (for Hippocampus only) (A+ defined as CSF Aβ_42_ < 980 pg/mL). Nominal *P*-values were adjusted for multiple testing using the Benjamini Hochberg procedure within the prespecified longitudinal biomarker analytic domain. BH-adjusted *P*-values < 0.05 are shown in bold. CSF, cerebrospinal fluid; AMPH, Amphiphysin-1; APOE, Apolipoprotein E; Aβ_42_, amyloid-β42; P-tau, phosphorylated-tau; T-tau, total-tau; A, amyloidosis.

### Interaction and subgroup analyses

No cross-sectional interaction remained significant after FDR correction (Supplementary Table 3). For longitudinal biomarkers, the association between baseline CSF AMPH and change in CSF P-tau was modified by sex (β = −0.031, *P* = 0.005, adjusted *P* = 0.017) and the association with longitudinal CSF T-tau change was also moderated by *APOE ε4* status (interaction β = −0.033, *P* = 0.002, adjusted *P* = 0.008) (Supplementary Table 4).

We therefore performed stratified analyses. In cross-sectional stratified models, CSF AMPH showed positive associations with CSF Aβ_42_, P-tau, and T-tau across all subgroups (Supplementary Table 5). In longitudinal subgroup analyses, higher baseline CSF AMPH was associated with decreasing CSF Aβ_42_ in CN participants, and among *APOE ε4* non-carriers, whereas significant associations with increases in CSF P-tau and T-tau were observed only among *APOE ε4* non-carriers (Supplementary Table 6).

### Association of CSF AMPH with cognitive performance and risk of clinical progression

**FIGURE 3:**
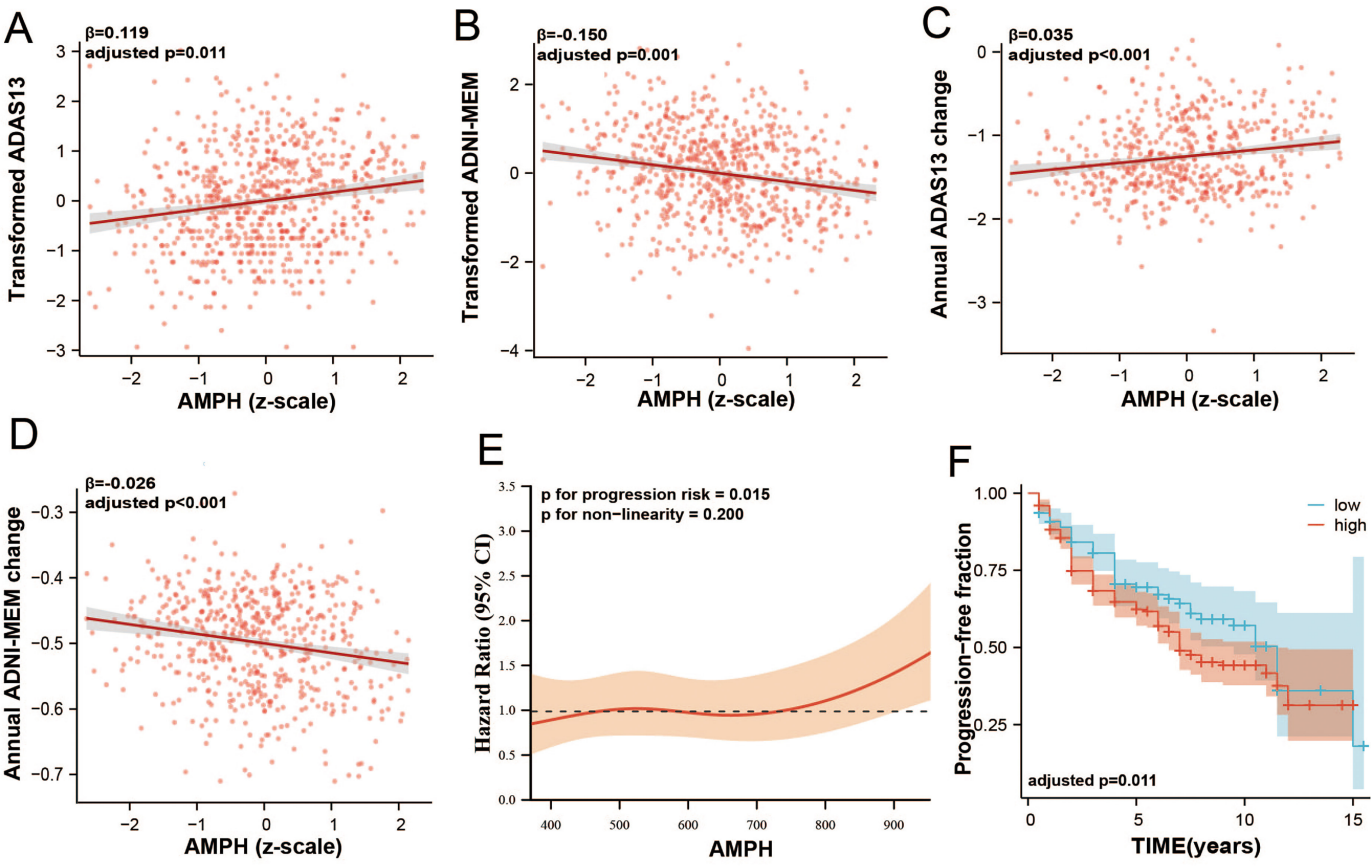
Cross-sectional and longitudinal associations of CSF AMPH with cognition and clinical progression. Baseline CSF AMPH was associated with baseline cognition assessed by ADAS-13 **(A)** and ADNI-MEM **(B)** and with longitudinal change in ADAS-13 **(C)** and ADNI-MEM **(D)**. Restricted cubic spline analysis of the association between baseline CSF AMPH and the risk of clinical progression. Hazard ratios were estimated using a Cox proportional hazards model adjusted for age, sex, education, *APOE ε4* status. The solid line represents the adjusted hazard ratio, and the shaded area represents the 95% confidence interval. **(E)**. Kaplan–Meier curves illustrate clinical progression in the overall cohort **(F)**. The normalized regression coefficients (β) and *P*-values were derived from multiple linear regression **(A,B)** and linear mixed-effects models **(C,D**) adjusted for age, sex, education, and *APOE ε4* status. Kaplan–Meier curves were compared using log-rank tests. CSF, cerebrospinal fluid; AMPH, amphiphysin-1; ADAS-13, Alzheimer’s Disease Assessment Scale–Cognitive Subscale 13; ADNI-MEM, ADNI memory composite score; MCI, mild cognitive impairment; APOE, Apolipoprotein E.

Cross-sectionally, higher baseline CSF AMPH levels were associated with worse global cognitive performance on ADAS-13 (β = 0.119, *P* = 0.003, adjusted *P* = 0.011) (Figure 3A), and poorer memory performance reflected by ADNI-MEM scores (β = −0.150, *P* < 0.001, adjusted *P* = 0.001) (Figure 3B). No significant cross-sectional associations were detected for ADAS-11, CDR-SB, MMSE, MoCA and executive function as measured by ADNI-EF (Supplementary Table 7). Longitudinally, higher baseline CSF AMPH levels were consistently associated with faster cognitive decline across multiple global and domain-specific measures, including ADAS-13 (β = 0.035, *P* < 0.001, adjusted *P* < 0.001), ADNI-MEM (β = −0.026, *P* < 0.001, adjusted *P* < 0.001) (Figures 3C, D), ADAS-11, CDR-SB, MMSE, MoCA and ADNI-EF (Supplementary Table 8). In Cox regression models, each 1-SD increase in CSF AMPH was linked to a 21% higher hazard of clinical progression (HR = 1.21, 95% CI: 1.05–1.37). Restricted cubic spline analysis further showed an overall association between baseline CSF AMPH and the risk of clinical progression, without evidence of statistically significant nonlinearity (P for overall association = 0.015; P for nonlinearity = 0.200) (Figure 3E). Consistent with these findings, Kaplan–Meier analyses indicated a higher risk of clinical progression among individuals with high baseline CSF AMPH in the overall cohort (*P* = 0.006, adjusted *P* = 0.011) (Figure 3F). In sensitivity analyses, the association remained evident using a median-based dichotomization (*P* = 0.021, adjusted *P* = 0.021) and quartile-based categorization (*P* = 0.007, adjusted *P* = 0.011) (Supplementary Figure 1). In the MCI subgroup, the analyses did not reach statistical significance (Supplementary Figure 2). Adding CSF Aβ_42_ and P-tau increased Nagelkerke pseudo R^2^ from 0.103 to 0.258. Adding CSF AMPH produced a small but statistically significant further increase to 0.267 (Supplementary Table 9).

## Discussion

In this study, we showed that (1) CSF AMPH was elevated in the AD group and showed variation across pathological stages; (2) higher CSF AMPH showed positive associations with CSF core AD biomarkers, and faster longitudinal decreases in CSF Aβ_42_, as well as with more rapid increases in CSF P-tau and T-tau among A− individuals; (3) higher baseline CSF AMPH predicted worse cognitive performance, steeper cognitive decline, and greater hippocampal atrophy, and was also associated with a higher risk of clinical progression. Prior evidence has shown that CSF AMPH levels are increased in patients with AD (Bergström et al., 2021). Our results extend this literature by demonstrating stage-dependent alterations in AMPH along the AT(N) continuum and by linking AMPH to longitudinal changes in core AD biomarkers, neuroimaging measures, cognition, and clinical progression. These findings may provide complementary information for understanding disease progression.

Consistent with prior reports, CSF AMPH levels were positively related to CSF Aβ_42_ across diagnostic groups (Bergström et al., 2021). One tentative explanation for this association relates to potential amyloid associated synaptic alterations. AMPH is involved in presynaptic vesicle cycling (Takei et al., 1999), and experimental data further show that Aβ impairs synaptic vesicle endocytosis and causes accumulation of AMPH at the plasma membrane of neurons (Kelly and Ferreira, 2007). Therefore, this positive association may reflect the downstream impact of amyloid pathology on synaptic integrity.

Moreover, CSF AMPH levels were elevated in T+ individuals and showed positive associations with CSF P-tau as well as T-tau, suggesting that AMPH might be linked to tau-related neurodegenerative processes (Cheng et al., 2025; De Jesús-Cortés et al., 2012; Remnestål et al., 2021). In A− participants, elevated baseline CSF AMPH was linked to greater declines in CSF Aβ_42_ over time and increases in CSF P-tau and T-tau. AMPH is a brain-enriched presynaptic protein concentrated at nerve terminals and implicated in synaptic vesicle cycling and synaptic signaling (Bauerfeind et al., 1997; Mundigl et al., 1998), and previous work has demonstrated that synaptic alterations are tightly coupled to extracellular Aβ dynamics and to tau accumulation and spreading (Ao et al., 2025; Cirrito et al., 2005; Franzmeier et al., 2024; Liu et al., 2012; Mattsson et al., 2013; Wu et al., 2016; Yamada et al., 2014). In this context, elevated CSF AMPH may index early synaptic alterations that foreshadow subsequent trajectories of core AD biomarkers in A− individuals. A plausible explanation for the lack of significant longitudinal associations in A+ individuals is stage-dependent biomarker kinetics: once amyloid positivity is established, CSF Aβ_42_ is often already reduced and may exhibit limited further decline, thereby restricting the dynamic range for detecting baseline predictors of Aβ_42_ slopes (Sutphen et al., 2015). At later disease stages, longitudinal tau trajectories may be driven more by amyloid-associated changes in soluble tau metabolism that precede aggregation and propagation, rather than by continued modulation by upstream synaptic variation (Mattsson-Carlgren et al., 2020; Pichet Binette et al., 2022). Moreover, CSF AMPH levels were lower in stage 1 individuals, such that a single baseline measurement may not fully capture synaptic status during follow-up and could attenuate associations with subsequent biomarker changes.

Notably, we found that CSF AMPH levels differed across AT(N) stages: specifically, reduced CSF AMPH levels were observed in A+ individuals with T− and N− status, whereas CSF AMPH levels were higher in the presence of tau pathology and/or neurodegeneration, even without A+. The mechanisms underlying the differed across AT(N) stages remain unclear, but several explanations can be proposed. In stage 1 participants, we observed lower biofluid AMPH, indicating that AMPH alterations can be detectable early in the Alzheimer’s disease continuum (Sperling et al., 2011). This pattern is compatible with CSF proteomic evidence showing that a subset of synapse-enriched proteins is reduced in preclinical AD stage 1, preceding changes in classical neurodegeneration markers (Ao et al., 2025; Goossens et al., 2023; Lleó et al., 2019). Mechanistically, AMPH is central to CME and works with dynamin to support synaptic vesicle retrieval and recycling (Jaye et al., 2024; McMahon and Boucrot, 2011; Wu et al., 2009). Experimental data further show that amyloid-β impairs synaptic vesicle endocytosis and causes accumulation of AMPH at the plasma membrane of neurons (Cao et al., 2010; Jaye et al., 2024; Kelly and Ferreira, 2007; Peter et al., 2004). Taken together, reduced CSF AMPH in stage 1 may reflect amyloid-associated early synaptic vesicle-cycle dysfunction and AMPH subcellular sequestration. In contrast, increased AMPH in later stages may reflect tau-driven neurodegeneration and increased release into the CSF. SNAP is a biomarker based concept denoting AD like neurodegeneration in individuals without β amyloidosis (Jack et al., 2016; Wisse et al., 2021). The highest CSF AMPH levels observed in the SNAP group indicate that AMPH elevation is not confined to amyloid positive AD pathophysiology. Together with recent evidence showing increased CSF AMPH across several neurodegenerative disorders (Mravinacová et al., 2025), this pattern suggests that CSF AMPH may be better characterized as a candidate marker of synaptic and neurodegenerative processes than as an AD specific biomarker. However, because these findings were derived from cross sectional comparisons across different individuals, they do not establish a within person biphasic trajectory. Serial within person CSF AMPH measurements are needed to determine whether the cross sectional stage differences reflect within person actual longitudinal trajectories.

To integrate our findings into the broader synaptic biomarker landscape, we briefly compare AMPH with other established CSF synaptic biomarkers. Neurogranin (Ng) is primarily associated with postsynaptic signaling and synaptic plasticity (Díez-Guerra, 2010), whereas synaptosome-associated protein (SNAP-25) is involved in presynaptic SNARE-mediated vesicle fusion and neurotransmitter release (Rizo and Xu, 2015), and growth-associated protein 43 (GAP-43) is associated with axonal growth and synaptic remodeling (Benowitz and Routtenberg, 1997). Previous studies have reported that CSF Ng, SNAP-25, and GAP-43 are elevated in AD or AD-related pathological profiles and are associated with tau-related pathology, cognitive impairment, and longitudinal clinical outcomes (Milà-Alomà et al., 2021; Portelius et al., 2015; Sandelius et al., 2019; Zhang et al., 2018). In comparison, AMPH is a presynaptic protein that interacts with dynamin and is involved in CME and synaptic-vesicle retrieval (Takei et al., 1999). In our cohort, CSF AMPH showed increased levels in AD and was associated with tau-related and neurodegenerative measures. Taken together, these findings suggest that CSF AMPH exhibits stage-dependent changes across the AD continuum, which are lower in stage 1 and elevated in stage 2 and SNAP, similar to that reported for other synaptic proteins (Goossens et al., 2023), which may help capture biologically distinct stages of disease. Importantly, we also found that high baseline AMPH levels were associated with faster hippocampal atrophy and a higher risk of clinical progression, suggesting that it might be relevant to AD related disease progression.

Several limitations should be noted. First, the precise biological mechanisms underlying these associations remain unclear. Future experimental studies, including cellular and animal models, will be needed to clarify whether and how alterations in AMPH are related to synaptic and neurodegenerative processes in disease. Second, a limitation of our study is the lack of orthogonal validation of the SomaScan derived CSF AMPH signal using alternative methods. Future studies employing immunoassays or targeted mass spectrometry may help confirm the observed associations. Besides, because the present analyses were conducted in a single research cohort, replication in independent cohorts would help determine the generalizability of the observed associations. Finally, the absence of comparison groups with other neurodegenerative disorders, such as frontotemporal dementia, dementia with Lewy bodies, progressive supranuclear palsy, and corticobasal syndrome, limited our ability to evaluate the biological significance of CSF AMPH elevation across neurodegenerative disorders or to determine its disease specificity. Therefore, the biological significance of elevated CSF AMPH across different neurodegenerative diseases, as well as its potential disease specificity, remains to be further investigated.

In summary, we evaluated the relationships between CSF AMPH and core AD biomarkers, neuroimaging indices, cognitive performance and clinical progression, which may provide complementary information for understanding disease worsening. Further studies are needed to evaluate the potential role of CSF AMPH as a marker of disease progression.

## Data Availability

The original contributions presented in this study are included in this article/Supplementary material, further inquiries can be directed to the corresponding authors.
