# Supplementary material for: Association of cerebrospinal fluid amphiphysin-1 levels with cognition and Alzheimer’s disease pathology biomarkers
Source: Front Aging Neurosci. 2026 Jul 21;18:1875646. doi: 10.3389/fnagi.2026.1875646 (PMC13433631; doi:10.3389/fnagi.2026.1875646)
Supplement: Supplementary file 1 [file Data_Sheet_1.docx]

**Supplementary Table 1 Box–Cox transformation parameters and Shapiro–Wilk test results**

| **Variable** | **Box–Cox λ** | **Shapiro-Wilk P before** | **Shapiro-Wilk P after** |
| --- | --- | --- | --- |
| CSF AMPH | −0.190 | <0.001 | 0.051 |
| CSF Aβ_42_ | 0.069 | <0.001 | <0.001 |
| CSF P-tau | -0.149 | <0.001 | 0.001 |
| CSF T-tau | -0.079 | <0.001 | 0.011 |
| Hippocampus | Not transformed | 0.068 | - |
| ADAS13 | 0.575 | <0.001 | 0.012 |
| ADAS11 | 0.461 | <0.001 | 0.004 |
| CDRSB | 0.187 | <0.001 | <0.001 |
| MMSE | 7.716 | <0.001 | <0.001 |
| MOCA | 1.701 | <0.001 | <0.001 |
| ADNI_MEM | Not transformed | 0.423 |  |
| ADNI_EF | 0.913 | 0.040 | 0.026 |

P values were computed with the Shapiro–Wilk test to assess normality.

Abbreviations: CSF, cerebrospinal fluid; AMPH, Amphiphysin-1; A, amyloidosis; T, tau pathology; ADAS-13, Alzheimer’s Disease Assessment Scale–Cognitive Subscale 13; ADAS-11, Alzheimer’s Disease Assessment Scale–Cognitive Subscale 11; CDR-SB, Clinical Dementia Rating–Sum of Boxes; MMSE, Mini–Mental State Examination; MoCA, Montreal Cognitive Assessment;

ADNI-MEM, ADNI memory composite score; ADNI-EF, ADNI executive function composite score

**Supplementary Table 2. Comparison of baseline CSF AMPH between groups in different populations.**

| **Measure** | **Transformed Mean** | **F value** | **P** | **Tukey HSD test** |
| --- | --- | --- | --- | --- |
| A- | -0.075 | 2.61 | 0.106 |  |
| A+ | 0.049 |  |  |  |
| T- | -0.657 | 462.42 | **< 0.001** |  |
| T+ | 0.597 |  |  |  |
| N- | -0.644 | 542.31 | **< 0.001** |  |
| N+ | 0.670 |  |  |  |
| Stage 0 | -0.526 | 186.42 | **< 0.001** | S0 - S1 **< 0.001**  S0 - S2 **< 0.001**  S0 - SNAP **< 0.001**  S1 - S2 **< 0.001**  S1 - SNAP **< 0.001**  S2 - SNAP **< 0.001** |
| Stage 1 | -0.899 |  |  |  |
| Stage 2 | 0.505 |  |  |  |
| SNAP | 0.903 |  |  |  |

P values were computed with the One-way ANOVA for comparison of means while Tukey HSD test was employed for post hoc test. Significant effects (P <0.05) are shown in bold.

Abbreviations: CSF, cerebrospinal fluid; AMPH, Amphiphysin-1; A, amyloidosis; T, tau pathology; N, neurodegeneration; SNAP, suspected non-Alzheimer’s pathophysiology; S0, stage 0; S1, stage 1; S2, stage 2.

| **Measure** | **Diagnosis** | | | **Sex** | | | ***APOE* ε4 status** | | |
| --- | --- | --- | --- | --- | --- | --- | --- | --- | --- |
|  | **β** | **P** | **Adjusted P** | **β** | **P** | **Adjusted P** | **β** | **P** | **Adjusted P** |
| CSF Aβ_42_ | -0.185 | 0.031 | 0.093 | 0.119 | 0.109 | 0.327 | -0.079 | 0.281 | 0.596 |
| CSF P-tau | 0.061 | 0.262 | 0.262 | -0.004 | 0.939 | 0.939 | -0.041 | 0.397 | 0.596 |
| CSF T-tau | 0.033 | 0.519 | 0.519 | −0.040 | 0.385 | 0.578 | 0.004 | 0.922 | 0.922 |

**Supplementary Table 3. Interaction analyses results of CSF AMPH by multiple linear regression model**

Adjusted for age, sex, education, baseline diagnosis, and *APOE ε4* status. BH adjusted P values P values < 0.05 are shown in bold.

Abbreviations: CSF, cerebrospinal fluid; AMPH, Amphiphysin-1; APOE, Apolipoprotein E; Aβ_42_, amyloid-β42; P-tau, phosphorylated-tau; T-tau, total-tau

**Supplementary Table 4. Interaction analyses results of CSF AMPH by linear mixed-effects models**

| **Measure** | **Diagnosis× time** | | | **Sex × time** | | | ***APOE* ε4 status× time** | | |
| --- | --- | --- | --- | --- | --- | --- | --- | --- | --- |
|  | **β** | **P** | **Adjusted P** | **β** | **P** | **Adjusted P** | **β** | **P** | **Adjusted P** |
| CSF Aβ_42_ | 0.012 | 0.358 | 0.358 | -0.017 | 0.191 | 0.191 | 0.021 | 0.101 | 0.101 |
| CSF P-tau | -0.019 | 0.087 | 0.139 | -0.031 | 0.005 | **0.017** | -0.024 | 0.035 | 0.054 |
| CSF T-tau | -0.019 | 0.074 | 0.113 | -0.021 | 0.055 | 0.091 | -0.033 | 0.002 | **0.008** |

Adjusted for age, sex, education, baseline diagnosis, and *APOE ε4* status. Nominal P values were adjusted for multiple testing using the Benjamini Hochberg procedure within the prespecified analytic domain. BH adjusted P values P values < 0.05 are shown in bold.

Abbreviations: CSF, cerebrospinal fluid; AMPH, Amphiphysin-1; APOE, Apolipoprotein E; Aβ_42_, amyloid-β42; P-tau, phosphorylated-tau; T-tau, total-tau

**Supplementary Table 5. Association between CSF AMPH and CSF biomarkers in different subgroups.**

| **Subgroup** | | **CSF Aβ_42_** | **CSF P-tau** | **CSF T-tau** |
| --- | --- | --- | --- | --- |
| CN | β | 0.351 | 0.694 | 0.758 |
|  | P | < 0.001 | < 0.001 | < 0.001 |
|  | **Adjusted P** | **< 0.001** | **< 0.001** | **< 0.001** |
| MCI | β | 0.146 | 0.769 | 0.801 |
|  | P | < 0.001 | < 0.001 | < 0.001 |
|  | **Adjusted P** | **< 0.001** | **< 0.001** | **< 0.001** |
| Female | β | 0.119 | 0.742 | 0.806 |
|  | P | 0.039 | < 0.001 | < 0.001 |
|  | **Adjusted P** | **0.039** | **< 0.001** | **< 0.001** |
| Male | β | 0.245 | 0.753 | 0.779 |
|  | P | < 0.001 | < 0.001 | < 0.001 |
|  | **Adjusted P** | **< 0.001** | **< 0.001** | **< 0.001** |
| *APOE ε4* (-) | β | 0.222 | 0.768 | 0.790 |
|  | P | < 0.001 | < 0.001 | < 0.001 |
|  | **Adjusted P** | **< 0.001** | **< 0.001** | **< 0.001** |
| *APOE ε4* (+) | β | 0.152 | 0.726 | 0.791 |
|  | P | 0.004 | < 0.001 | < 0.001 |
|  | **Adjusted P** | **0.004** | **< 0.001** | **< 0.001** |

The normalized regression coefficients (β) and P values were derived from multivariable linear regression models adjusted for age, sex, education, baseline diagnosis, and *APOE ε4* status. Nominal P values were adjusted for multiple testing using the Benjamini Hochberg procedure within the prespecified analytic domain. BH adjusted P values P values < 0.05 are shown in bold.

Abbreviations: CSF, cerebrospinal fluid; AMPH, Amphiphysin-1; APOE, Apolipoprotein E; Aβ_42_, amyloid-β42; P-tau, phosphorylated-tau; T-tau, total-tau

**Supplementary Table 6. Linear mixed-effects models for baseline CSF AMPH and longitudinal CSF biomarkers in different subgroup**

| **Subgroup** | | **CSF Aβ_42_** | **CSF P-tau** | **CSF T-tau** |
| --- | --- | --- | --- | --- |
| CN | β | -0.021 | 0.016 | 0.014 |
|  | P | 0.008 | 0.078 | 0.076 |
|  | **Adjusted P** | **0.026** | 0.078 | 0.078 |
| MCI | β | -0.011 | -0.005 | -0.004 |
|  | P | 0.241 | 0.4332 | 0.539 |
|  | **Adjusted P** | 0.539 | 0.539 | 0.539 |
| Female | β | -0.004 | 0.014 | 0.012 |
|  | P | 0.685 | 0.083 | 0.083 |
|  | **Adjusted P** | 0.685 | 0.125 | 0.125 |
| Male | β | -0.019 | -0.004 | -0.004 |
|  | P | 0.019 | 0.602 | 0.609 |
|  | **Adjusted P** | 0.056 | 0.609 | 0.609 |
| *APOE ε4*(-) | β | -0.022 | 0.019 | 0.021 |
|  | P | 0.008 | 0.004 | < 0.001 |
|  | **Adjusted P** | **0.008** | **0.006** | **0.002** |
| *APOE ε4*(+) | β | -0.005 | -0.015 | 0.014 |
|  | P | 0.696 | 0.172 | 0.154 |
|  | **Adjusted P** | 0.696 | 0.259 | 0.259 |

The normalized regression coefficients (β) and P values were derived from the interaction term of CSF AMPH × time in the linear mixed-effects models adjusted for age, sex, education, baseline diagnosis, and *APOE ε4* status. Nominal P values were adjusted for multiple testing using the Benjamini Hochberg procedure within the prespecified analytic domain. BH adjusted P values P values < 0.05 are shown in bold.

Abbreviations: CSF, cerebrospinal fluid; AMPH, Amphiphysin-1; APOE, Apolipoprotein E; Aβ_42_, amyloid-β42; P-tau, phosphorylated-tau; T-tau, total-tau

**Supplementary Table 7. Association between CSF AMPH and cognitive**

| Cognition | CSF AMPH | | |
| --- | --- | --- | --- |
|  | **β** | **P value** | **Adjusted P** |
| ADAS-13 | 0.119 | 0.003 | **0.011** |
| ADAS-11 | 0.090 | 0.028 | 0.066 |
| CDR-SB | 0.047 | 0.245 | 0.253 |
| MMSE | -0.051 | 0.205 | 0.253 |
| MoCA | -0.087 | 0.069 | 0.121 |
| ADNI-MEM | -0.150 | < 0.001 | **0.001** |
| ADNI-EF | -0.045 | 0.253 | 0.253 |

The normalized regression coefficients (β) and P values were derived from multivariable linear regression models adjusted for age, sex, education, and *APOE ε4* status. Nominal P values were adjusted for multiple testing using the Benjamini Hochberg procedure within the prespecified analytic domain. BH adjusted P values P values < 0.05 are shown in bold.

Abbreviations: CSF, cerebrospinal fluid; AMPH, Amphiphysin-1; APOE, Apolipoprotein E; ADAS-13, Alzheimer’s Disease Assessment Scale–Cognitive Subscale 13; ADAS-11, Alzheimer’s Disease Assessment Scale–Cognitive Subscale 11; CDR-SB, Clinical Dementia Rating–Sum of Boxes; MMSE, Mini–Mental State Examination; MoCA, Montreal Cognitive Assessment;

ADNI-MEM, ADNI memory composite score; ADNI-EF, ADNI executive function composite score

**Supplementary Table 8. Linear mixed-effects models for baseline CSF AMPH and longitudinal cognitive change**

| Cognition | CSF AMPH | | |
| --- | --- | --- | --- |
|  | **β** | **P value** | **Adjusted P** |
| ADAS-13 | 0.035 | < 0.001 | **< 0.001** |
| ADAS-11 | 0.045 | < 0.001 | **< 0.001** |
| CDR-SB | 0.053 | < 0.001 | **< 0.001** |
| MMSE | -0.054 | < 0.001 | **< 0.001** |
| MoCA | -0.023 | 0.013 | **0.013** |
| ADNI-MEM | -0.026 | < 0.001 | **< 0.001** |
| ADNI-EF | -0.019 | 0.008 | **0.009** |

The normalized regression coefficients (β) and P values were derived from the interaction term of CSF AMPH × time in the linear mixed-effects models adjusted for age, sex, education, and *APOE ε4* status. Nominal P values were adjusted for multiple testing using the Benjamini Hochberg procedure within the prespecified analytic domain. BH adjusted P values P values < 0.05 are shown in bold.

Abbreviations: CSF, cerebrospinal fluid; AMPH, Amphiphysin-1; APOE, Apolipoprotein E; Aβ42, amyloid-β42; ADAS-13, Alzheimer’s Disease Assessment Scale–Cognitive Subscale 13; ADAS-11, Alzheimer’s Disease Assessment Scale–Cognitive Subscale 11; CDR-SB, Clinical Dementia Rating–Sum of Boxes; MMSE, Mini–Mental State Examination; MoCA, Montreal Cognitive Assessment; ADNI-MEM, ADNI memory composite score; ADNI-EF, ADNI executive function composite score.

**Supplementary Table 9 Incremental prognostic value of CSF AMPH for clinical progression in nested Cox proportional hazards models.**

| **Model** | **Nagelkerke R²** | **LRT P value** |
| --- | --- | --- |
| Model 1: age, sex, education, diagnosis, and *APOE ε4* | 0.103 |  |
| Model 2: Model 1 + CSF Aβ42 + CSF P-tau | 0.258 | **< 0.001** |
| Model 3: Model 2 + CSF AMPH | 0.267 | **0.011** |

Notes: Nested Cox proportional hazards models were fitted for clinical progression in the complete case sample. Model 1 included age, sex, education, baseline diagnosis, and *APOE ε4* status. Model 2 additionally included CSF Aβ42 and P-tau, and Model 3 further included CSF AMPH. Model fit was assessed using Nagelkerke pseudo R².

Abbreviations: CSF, cerebrospinal fluid; AMPH, Amphiphysin-1; APOE, Apolipoprotein E; Aβ_42_, amyloid-β42; P-tau, phosphorylated-tau; T-tau, total-tau

**Supplementary Figure 1. Kaplan–Meier curves for clinical progression by baseline CSF AMPH level**

Nominal P values were adjusted for multiple testing using the Benjamini Hochberg procedure within the prespecified analytic domain.

Abbreviations: CSF, cerebrospinal fluid; AMPH, Amphiphysin-1;

**Supplementary Figure 2. Kaplan–Meier Curves for Clinical Progression by Baseline CSF AMPH Level in MCI group.**

Nominal P values were adjusted for multiple testing using the Benjamini Hochberg procedure within the prespecified analytic domain.

Abbreviations: CSF, cerebrospinal fluid; AMPH, Amphiphysin-1.
